# Circum-Saharan Prehistory through the Lens of mtDNA Diversity

**DOI:** 10.3390/genes13030533

**Published:** 2022-03-17

**Authors:** Mame Yoro Diallo, Martina Čížková, Iva Kulichová, Eliška Podgorná, Edita Priehodová, Jana Nováčková, Veronica Fernandes, Luísa Pereira, Viktor Černý

**Affiliations:** 1 Archaeogenetics Laboratory, Institute of Archaeology of the Academy of Sciences of the Czech Republic, Letenská 1, 118 01 Prague, Czech Republic; yorodiallo90@yahoo.fr (M.Y.D.); martina.ciz@email.cz (M.Č.); ell.6@email.cz (E.P.); priehodovaedita@seznam.cz (E.P.); 2 Department of Anthropology and Human Genetics, Faculty of Science, Charles University in Prague, 128 01 Prague, Czech Republic; iwakulichova@gmail.com; 3 Department of Forensic Genetics, Institute of Criminalistics, Police of the Czech Republic, 128 01 Prague, Czech Republic; novackova.janka@gmail.com; 4 i3S—Instituto de Investigação e Inovação em Saúde, Universidade do Porto, 4200-135 Porto, Portugal; vfernandes@ipatimup.pt (V.F.); luisap@ipatimup.pt (L.P.); 5 IPATIMUP—Instituto de Patologia e Imunologia Molecular da Universidade do Porto, 4200-135 Porto, Portugal

**Keywords:** Sahel/Savannah belt, North Africa, mtDNA diversity, population history

## Abstract

African history has been significantly influenced by the Sahara, which has represented a barrier for migrations of all living beings, including humans. Major exceptions were the gene flow events that took place between North African and sub-Saharan populations during the so-called African Humid Periods, especially in the Early Holocene (11.5 to 5.5 thousand years ago), and more recently in connection with trans-Saharan commercial routes. In this study, we describe mitochondrial DNA (mtDNA) diversity of human populations from both sides of the Sahara Desert, i.e., both from North Africa and the Sahel/Savannah belt. The final dataset of 7213 mtDNA sequences from 134 African populations encompasses 470 newly collected and 6743 previously published samples, which were analyzed using descriptive methods and Bayesian statistics. We completely sequenced 26 mtDNAs from sub-Saharan samples belonging to the Eurasian haplogroup N1. Analyses of these N1 mitogenomes revealed their possible routes to the Sahel, mostly via Bab el-Mandab. Our results indicate that maternal gene flow must have been important in this circum-Saharan space, not only within North Africa and the Sahel/Savannah belt but also between these two regions.

## 1. Introduction

The out-of-Africa event, during which a relatively small group of anatomically modern humans spread from East Africa into Eurasia [1], was a defining moment in the evolution of modern humans. Although paleoanthropology has detected several older waves of Middle Pleistocene migrations from Africa to Eurasia [2], genetic studies show that contemporary non-sub-Saharans are descendants of an ancestral population that spread from Africa only about 60 ka (thousands of years ago) [3]. Leaving aside the long-term isolation of Khoisan populations in southern Africa and of the Pygmies in the tropical rain forests of central Africa, the out-of-Africa event is nowadays considered to be the most significant restriction of gene flow between two groups of anatomically modern humans: the sub-Saharans and non-sub-Saharans had been separated by the Sahara Desert throughout most of prehistory. Differentiation between these groups is apparent in both mitochondrial (mtDNA) [4] and nuclear [5] DNA diversity. Due to this separation, we can detect different mtDNA haplogroups, which can be assigned either a sub-Saharan or Eurasian ancestry [6]. While the basis of the sub-Saharan mtDNA gene pool is classified as macro-haplogroup L, the rest of the world nowadays traces its maternal ancestry from haplogroup N or M [7].

That, however, does not mean that after the out-of-Africa event migration had stopped. The genetic structure of inhabitants of the Sahel/Savannah belt was analyzed by researchers with respect to their linguistic affiliation, subsistence structure, and geographic localization of local populations [8,9,10], and both population genetics and phylogeographic studies highlighted the significance of gene flow. The Sahel/Savannah belt has therefore been called a “bidirectional corridor of migrations” [11] and evidence of gene flow was also detected across the Sahara in populations inhabiting regions between the Sahel/Savannah belt and North Africa [12,13].

Interestingly, while some migrations may have had an ethnic association, others did not. For instance, the origin of the Chadic-speaking peoples living in the Lake Chad Basin was traced to East Africa based on linguistic evidence [14]. According to this theory, the ancestors of current Chadic-speaking peoples migrated, still as nomadic herders, from the Nile Valley through Wadi Howar to the Ennedi Mountains, and further through Wadi Hawash up to the Lake Chad Basin. Genetically, the Chadic-speaking peoples nowadays harbor mtDNA sequences belonging to the L3f haplogroup with East African ancestry, especially a private branch L3f3, which formed during their westward expansion of about 8 ka [15]. On the other hand, another mtDNA haplogroup, called L3e5, which was also detected in populations living today in the Lake Chad Basin but not only in Chadic-speaking populations, is also present in the Maghreb and its origin can be traced to an ancestral population that crossed the green Sahara during the Early Holocene approximately 10 ka [13].

Sahelian populations also carry Eurasian mtDNA haplogroups. They are found more frequently in the nomadic pastoralists than in sedentary farmers [16] and a surprising finding showed that some sub-Saharan Africans and even Northern Eurasians share some very recent maternal ancestry. For instance, it was shown that a Saami from Scandinavia and a Yakut from Siberia share with a Berber and a Fulani mtDNA sequences belonging to haplogroup U5b1b [17]. Given the enormous geographical distances between these populations, the most plausible explanation is that the most recent common ancestor (~8.6 ka) lived probably in southwestern Europe, from where the descendants spread both to northern Eurasia and sub-Saharan Africa. A more detailed study of originally Eurasian lineages beyond the Sahara has shown that not only U5b1b but also the H1 haplogroup (which both occur mainly in the Fulani pastoralists) came to form the new and younger sub-Saharan lineages called U5b1b1b and H1cb1 [18]. Their most recent common ancestor (~4 ka) dates to the time when, according to archaeology [19,20,21], the first herders settled in the western Sahel/Savannah belt.

It may therefore seem that the pastoralist food-production strategy did not spread to sub-Saharan Africa by demic diffusion from the Near Eastern domestication center via northeastern Africa, but through the ancestors of Berbers from the Maghreb. In this context, it should be noted that the genetic architecture of the circum-Mediterranean space had undergone substantial changes since the Neolithic. For instance, ancient Near Eastern farmers are genetically better represented by the current populations of central and western Mediterranean, such as the Sardinians and the Basques [22,23], than by the current populations of the Near East.

The importance of post-Neolithic gene flow from northwestern Africa to the western part of the Sahel/Savannah was also suggested by research on lactase persistence. In fact, the Fulani pastoralists from Burkina Faso share with Europeans the extended haplotype carrying Eurasian variant −13,910 × T. It was suggested that their ancestors received this haplotype via admixture with the Eurasian population two times [24]. The first event is genetically dated to ~1828 years ago and the second one to ~302 years ago, whereby it seems that the admixture involved a group related to southwestern Europeans. Moreover, the geographical distribution of lactase persistence variants in the Sahel/Savannah belt shows clear differences between the pastoralists in the east (mostly Arabs harboring variant −13,915 × G) and the west (mostly Fulani harboring variant −13,910 × T) [25]. In fact, a boundary between the western and eastern Sahelian genetic spaces lies somewhere near the Lake Chad Basin, as attested not only by lactase persistence but also by a genome-wide SNP study [26].

Last but not least, it was shown that Sahelian pastoralists tend to represent several mutually similar mtDNA haplotypes, which indicates either more recent origins of their diversity, isolation of their demes, lower gene flow, or lower effective size of the population [10]. Interestingly, thanks to coalescence analyses, it was possible to show there is an asymmetric gene flow between the pastoralists and the farmers in both parts of the Sahel/Savannah belt: while the western (Fulani) pastoralists are losing their mtDNA diversity, the eastern (Arabs) pastoralists are gaining it by admixture with local sub-Saharan agricultural populations [27]. This is further supported by the presence of various sub-Saharan mtDNA haplotypes in the gene pool of Arabic-speaking populations [9], mostly non-carriers of the lactase persistence −13,915 × G variant [28]. Interestingly, this genetic observation might correspond to a process of Arabization and/or language shift after the expansion of Arabs and their culture from North Africa into the Lake Chad Basin, from the 14th century AD onwards [29].

The above-mentioned studies show that inclusion of newly collected local populations, especially from the Sahel/Savannah belt, has significantly contributed to our knowledge of the peopling of Africa north of the equator by discoveries of not only new variants—which happens quite commonly when a new dataset of a sub-Saharan population is presented [30]—but even of entire new mtDNA haplogroups. In fact, since all new sub-Saharan population studies published so far revealed new genetic variants, one ought to admit we are so far aware of merely a fraction of the genetic diversity of sub-Saharan populations [31,32].

Because sub-Saharan Africa is still underrepresented in population genetic and genomic studies [30], we compiled a large mtDNA database composed of both newly collected and previously published mtDNA sequences and produced an updated survey of migration patterns in the circum-Saharan space. Additionally, we performed a complete mtDNA sequencing of the N1 haplogroup from sub-Saharan Africa, with most samples from the Sahel/Savannah belt but some also from East Africa. The N1 haplogroup’s southwestern Asian ancestry is well known and goes as far as to ~60 ka [4] but its African phylogeny is still not well understood. We selected the N1 because this haplogroup was reintroduced back to the Sahel/Savannah belt by migration from southwestern Asia, possibly via North Africa, as became apparent when a related basal branch was recently discovered in a North-African skeleton (Takarkori rock shelter, Libya) dated to ~7 ka [33]. We can thus assume that phylogeny of this specific haplogroup could document an ancient gene flow back to Africa in the eastern circum-Saharan region.

## 2. Materials and Methods

We built a large dataset of 7213 mtDNA hypervariable segment 1 (HVS-1) sequences from 134 African populations inhabiting the circum-Saharan space. It contains both published and newly acquired samples. All samples were collected with the help of African colleagues and appropriate informed consent was procured prior to sampling from all participants. Newly collected and here for the first time presented mtDNA (*n* = 470) sequences enriched the Sahelian dataset with four populations from Mali (*n* = 135), three from Mauritania (*n* = 191), and three from Sudan (*n* = 144). During the sampling, we focused on populations that have not been previously included in any genetic study, such as the Bella from Mali, Imraghen from Mauritania, Daju and Zaghawa from Sudan, but also on populations whose sampling coverage was insufficient due to their large geographic dispersals, such as the Fulani from Mali and Mauritania, Moors and Soninke from Mauritania, and Tuareg, Songhai, and Arabs from Mali.

**Figure 1:**
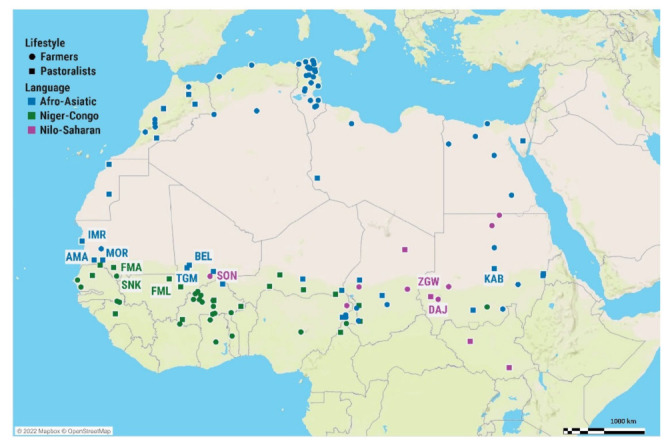
Geographic locations of 134 African populations included in this study. Newly obtained samples are marked with labels, and colors differentiate the linguistic affiliations and subsistence strategies.

The population dataset (Supplementary Table S1) was further divided according to three variables: from the perspective of subsistence strategy (lifestyle), each population was categorized as belonging either among pastoralists or farmers, from the perspective of language affiliation, populations were classified as belonging to one of three language families (Niger-Congo, Nilo-Saharan, and Afro-Asiatic), and from the geographic perspective we divided populations in western Sahel/Savanah, western North African, eastern Sahel/Savannah, and eastern North African groups (Figure 1).

Subsequently, we selected from our collections for complete mtDNA sequencing 26 DNA samples which, according to HVS-1 using the rCRS-oriented version of Build 17 on the PhyloTree website [34], belong (together with its daughter clade I) to haplogroup N1. For selected details of the N1 sample, see Supplementary Table S2. Subsequently, we compared these mitogenomes with worldwide samples (*n* = 701) belonging to the same N1 haplogroup. Our total N1 dataset thus includes 727 mitogenomes (Supplementary Table S3).

New samples published here for the first time were collected by the Oragene DNA Collection saliva kit (DNA Genotek Inc., Ottawa, ON, Canada). DNA was extracted according to supplier’s protocol. PCR amplification of all newly sequenced samples (*n* = 470) was carried out using primer pairs previously published in Gonder et al. [35]. Due to differences in the lengths of mtDNA sequences published in available comparative studies, sequences analyzed in this study were restricted to a highly polymorphic segment of 339 bp (nps 16,032–16,370). The region between positions 16,184 and 16,194 was excluded from analyses because of uncertainty regarding its correct alignment which arose in consequence of heteroplasmy observed in several samples [36].

Samples selected for whole mtDNA sequencing were analyzed using the Massive Parallel Sequencing (MPS) method. Quantity of the extracted DNA was determined by Plexor HY (Promega) and the samples were diluted to the total DNA template of 15 ng per reaction. Whole-genome mtDNA MPS data were generated using commercially available QIAseqTM Targeted DNA Panel (Qiagen), which contains 222 overlapping primers, and then sequenced on MiSeq Fgx Sequencing system (Verogen) in the “Research Use Only” mode through the “Generate FASTQ” with “FASTQ Only” application and “Paired End Read”. MPS libraries and final sequencing reactions were prepared according to the manufacturer’s protocol to a final loading concentration of 12 pM. Sequencing reaction was performed using MiSeq v2 Reagent Illumina kit (300 cycles per kit).

We computed both the standard and molecular diversity indices, such as haplotype diversity, nucleotide diversity, and the mean number of pairwise differences for each population using Arlequin software ver. 3.5.2.2 [37]. Differences in average haplotype and nucleotide diversities and in the mean number of pairwise differences among geographically specified subsistence modes were tested using Kruskal–Wallis one-way tests.

To investigate the demographic history of our samples, we calculated tests of selective neutrality such as Tajima’s D [38], Fu’s Fs [39], and R2 [40], which are sensitive to deviations from a demographic equilibrium. Significant results could indicate not only natural selection but also demographic expansion or contraction of effective population size. Tajima’s D and Fu’s Fs tests were calculated in the same version of Arlequin using 10,000 iterations [37], while R2 tests were computed by using DnaSP ver. 6.12.03 [41]. Statistical tests and confidence intervals for R2 were based on parametric bootstrapping with coalescence simulations. It should be noted that in order to reject neutrality at 5% level of significance in Fu’s Fs statistic, the *p*-value should be below 2% (because it is tested by a unilateral test). Additionally, we evaluated demographic history by Harpending’s raggedness index (Hri) and the sum of squared deviations (SSD) while considering a model of demographic expansion as implemented in Arlequin [42]. Statistical significance of values was assessed by a permutation test with 1000 replicates and, similarly, as in molecular diversity indices, variance between *p*-values was evaluated using Kruskal–Wallis one-way tests.

Interpopulation comparisons were assessed via pairwise Reynolds’ genetic distances based on haplotype frequencies [43,44] using Arlequin, and significance was tested by 10,000 iterations. Evolutionary distances between haplotypes (ΦST indices) were used to weight Reynolds’ genetic distances, to which purpose we used the 2-parameter Kimura model with a γ shape parameter of 0.4, transition/transversion ratio of 10/1, and indels not taken into consideration, as recommended in a previous study [45]. Coancestry coefficients of Reynolds’ genetic distances were used for visualization via a multidimensional scaling analysis (MDS) with community ecology package vegan ver. 2.5-7 [46] implemented in RStudio [47].

Analyses of molecular variance (AMOVA) assessed the levels of genetic variation within and between different groups of populations structured, as noted above, by several factors, namely language affiliation, geographical location, and subsistence strategy. The settings were: language grouping (Afro-Asiatic vs. Nilo-Saharan vs. Niger-Congo); geographic grouping as Region1 (Sahel/Savannah belt vs. North Africa) and Region2 (eastern North Africa vs. western North Africa vs. eastern Sahel/Savannah belt vs. western Sahel/Savannah belt), subsistence grouping as Lifestyle1 (pastoralists vs. farmers) and Lifestyle2 (pastoralists of the Sahel/Savannah belt vs. pastoralists of North Africa vs. farmers of Sahel/Savannah belt vs. farmers of North Africa). By inspecting the fixation indices, we were able to describe the structure between groups (for Φ_CT_ index) and/or relationships between populations within groups (for Φ_SC_ index). The significance of fixation indices was tested using a hierarchical framework and 10,000 iterations of the random permutation procedure implemented in Arlequin [37].

Coalescent estimation of ancestral or contemporary migration patterns between populations was carried out by Migrate-n (version 3.6.11) using a Bayesian Markov chain Monte Carlo inference model to generate a posterior probability density distribution [48]. This method is capable of measuring complex models with asymmetric gene flow directly. The estimated parameters were Θ for population size (from which we could calculate effective population size via Θ = 2Neµ for haploid mtDNA data) and M (immigration rates), both scaled to mutation rate, with settings of one long chain; 1,000,000 genealogies with every 5000 recorded; and a burn-in per replicate of 10,000. To enhance our understanding of the complex patterns and trends in gene flows, we used a bilateral circular migration plot as an effective method of visualizing flow data, implemented in the R package circlize 0.4.13 [49].

In terms of treatment of whole mtDNA sequences, we analyzed the raw FASTQ files using NextGENe software (SoftGenetics, LLC, PA 16803, USA) and the resulting sequences were read with BioEdit version 7.0.9.1 [50]. Mutations were scored relative to the revised reference sequence, rCRS [51] with numbers 1–16,569 referring to mutation’s position in that sequence. The newly generated mitogenomes were compared to complete sequences available in GenBank. We found 701 sequences belonging to I and N1 haplogroups in a great majority of cases from non-African populations.

To estimate time to the most recent common ancestor (TMRCA) for specific clades in the phylogeny, we used the ρ statistic [52] and maximum likelihood (ML). For ρ, i.e., the mean sequence divergence from the inferred ancestral haplotype of the clade in question, we set the mutation rate estimate for the whole-mtDNA sequence corrected for purifying selection to one substitution in every 3624 years and for the synonymous mutation rate at one substitution in every 7884 years [53], while standard errors were estimated as previously described [54]. ML estimates of branch lengths were obtained using PAML 4.8a [55] while assuming an HKY85 mutation model with γ-distributed rates (approximated by a discrete distribution with 32 categories), partitions (the two hypervariable regions as a block vs. the remaining mtDNA genome), and a generation time of 25 years. We converted mutational distance in ML to time using the same whole-mtDNA genome clock of 3624 years.

## 3. Results

**Figure 2:**
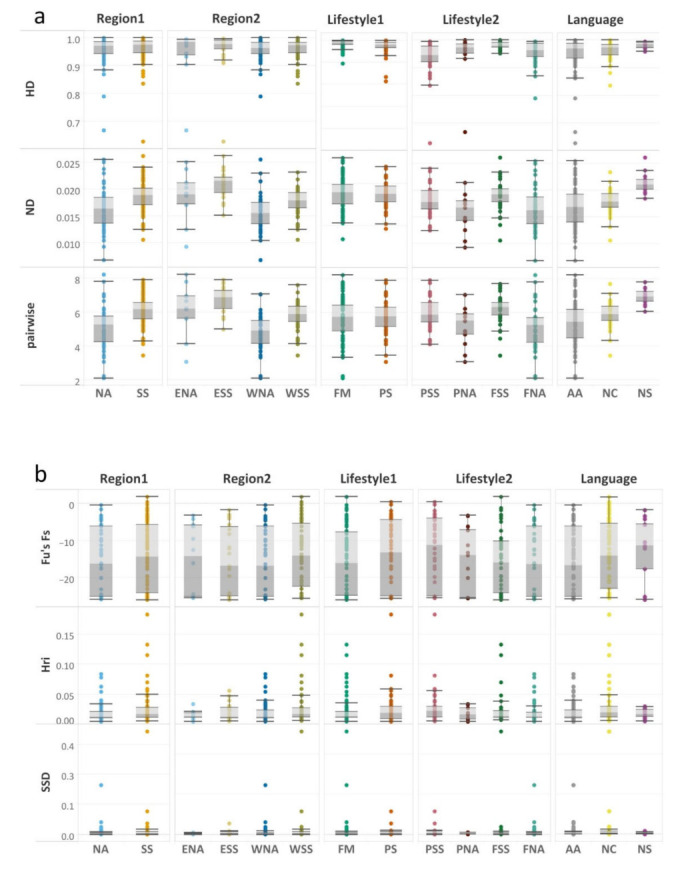
Boxplots representing diversity values of basic standard and molecular indices in five groups defined by subsistence strategy, language, and geographic region. (**a**) HD—haplotype diversity, ND—nucleotide diversity; pairwise—the mean number of pairwise differences; (**b**): Fs—Fu’s; Hri–Harpending’s raggedness index; SSD—the sum of squared deviations. Grouping label abbreviations: Region1: NA North Africa, SS Sahel/Savannah belt, Region2: ENA Eastern North Africa, ESS Eastern Sahel/Savannah belt, WNA Western North Africa, WSS Western Sahel/Savannah belt. Lifestyle1: PS Pastoralists, FM Farmers, Lifestyle2: PSS pastoralists of Sahel/Savannah belt, PNA pastoralists of North Africa, FSS farmers of Sahel/Savannah belt, FNA farmers of North Africa. Language: AA Afro-Asiatic, NC Niger-Congo, NS Nilo-Saharan. The color palette is set according to the defined groups. The line dividing boxplots into two parts represents the median of data for the variable concerned. The ends of the box indicate the upper (Q3) and lower (Q1) quartiles. The difference between quartiles 1 in light grey and 3 in grey indicates the interquartile range (IQR). The top and bottom lines show the maximum and minimum values. Dots (or other markers) beyond the extreme line indicate potential outliers.

Gene diversity, nucleotide diversity, and the mean number of pairwise differences of each population are shown in Supplementary Table S4. Figure 2a shows the distribution of values of these indices within geographical regions, subsistence categories, and language families. These results clearly show that populations from the Sahel/Savannah belt tend to be more diverse than those living in North Africa and that the eastern parts of both of these regions are more diverse than their western parts. One can also observe that sedentary farmers are slightly more diverse than nomadic pastoralists are, especially in the eastern part of the investigated regions. Our results regarding the distribution of these indices within language families may be affected by unequal representation of individual populations in groups because the Afro-Asiatic family is much better represented than other families: for instance, the Nilo-Saharan family is represented in our dataset by just 14 populations. A Kruskal–Wallis one-way test (Table 1) indicated statistically significant differences from both the perspective of nucleotide diversity and the mean number of pairwise differences among all the groups (except for those based on lifestyle, which seems to significantly differ from the perspective of haplotype diversity). When lifestyle was combined with regional information, all molecular indices showed significant differences.

Patterns of demographic expansions were investigated using selective neutrality tests. Figure 2b shows the distribution of values of these indices within geographical regions, subsistence categories, and languages families. A more sensitive analysis—indicated by the highly negative values of Fu’s Fs (running around −25) supported by non-significant *p*-values of SSD and Hri—revealed demographic expansion in several North African populations. As far as sedentary farmers are concerned, demographic contraction can be deduced for the Bedik and Mandenka, but also for some groups of Moroccan Berbers such as the Kesra and Zriba. No signals of demographic expansion (based on Fu’s Fs) were detected also in several nomadic Fulani groups from the western Sahel/Savannah belt. The exact values of all indices are specified in Supplementary Table S4. We also tested the variance of *p*-values with a Kruskal–Wallis one-way test and significant differences were detected only in the Hri index between linguistic groups (Table 1).

**Figure 3:**
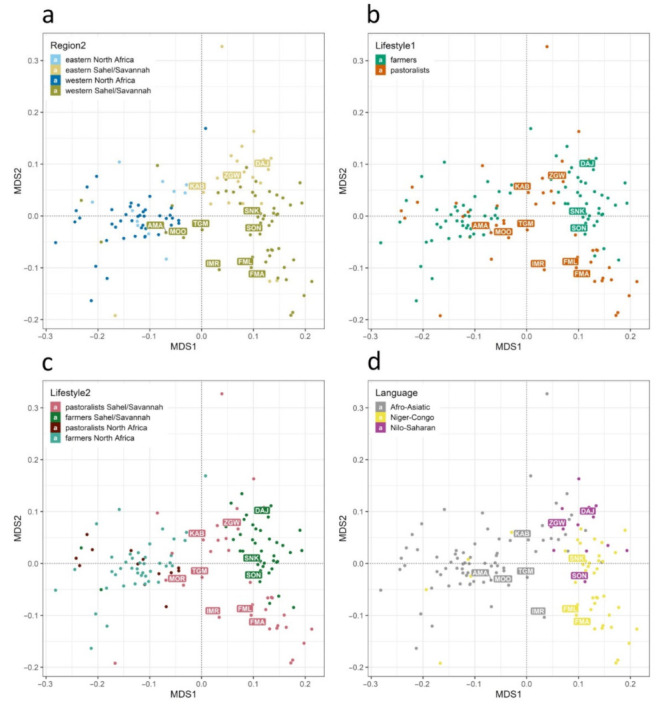
MDS plot (stress value 0.112) of pairwise mtDNA genetic distances between 134 populations colored according to geography as Region2 (**a**), subsistence as Lifestyle1 (**b**) and Lifestyle2 (**c**), and Language (**d**). Only newly collected populations are highlighted with labels.

A matrix of coancestry coefficients for all analyzed populations is presented in Supplementary Table S5. MDS plots based on these values (calculated from genetic distances by −ln(1 − F_ST_)) with a stress value of 0.112 imply differentiation of populations in a 2D scale. Four subplots show the populations colored according to geography (Figure 3a), lifestyle (Figure 3b,c), and language (Figure 3e). In general, the space covered by populations from the Sahel/Savannah belt is apparently larger than the space covered by the North African populations, which shows a higher level of differentiation (higher pairwise distances) in the southern part of this circum-Saharan area. Interestingly, the newly introduced populations collected in the Sahel/Savannah belt are distributed not only within their own genetic space: some, such as the Moors from Mauritania and Arabs from Mali, are located within the area covered by North African populations. This can best be explained by the recent immigration of these groups to the Sahel/Savannah belt. Figure 3d shows that not only geography but also language might be considered a good tool for the determination of population structure in the circum-Saharan space.

**Figure 4:**
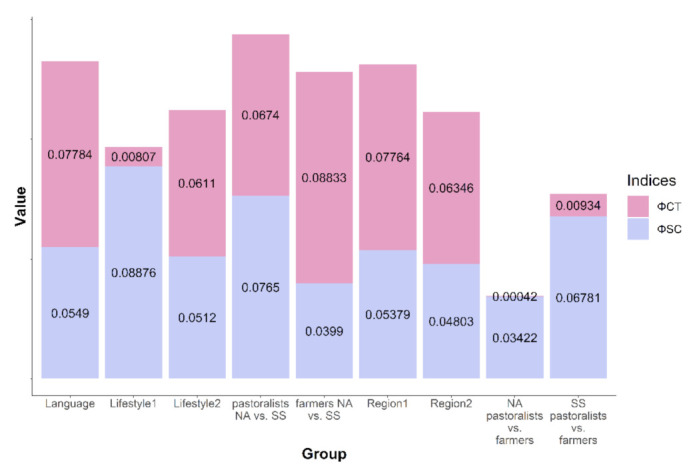
Values of two variation indices Φ_CT_ in pink (for variation between groups) and Φ_SC_ in violet (for variation among populations within the groups) from analysis of molecular variance (AMOVA) made for several groups defined by language, lifestyle, and geographic region (groups specified on *x*-axis).

AMOVA was applied to evaluate genetic differentiation among populations grouped according to geography, language, and subsistence strategy. The results are presented in Supplementary Table S6 and Figure 4. Interestingly, definitions of the groups based on lifestyle do not seem to reflect the population structure either in North Africa or in the Sahel/Savannah belt: in all these groups, the values for between-group variation (Φ_CT_) were lower than for variation between populations within these groups (Φ_SC_). On the other hand, we were able to confirm the structure when considering languages or regionally divided populations (Region1 and Region2), or when the farmers/pastoralists in North Africa were contrasted with farmers in the Sahel/Savannah belt. Our results show rather balanced indices indicating no significant structure among pastoralists in North Africa and the Sahel/Savannah belt (Φ_CT_ = 0.0674, Φ_SC_ = 0.0765). All values were statistically highly significant (*p* < 0.05) except that for variance between the groups of farmers vs. pastoralists in the North African division.

**Figure 5:**
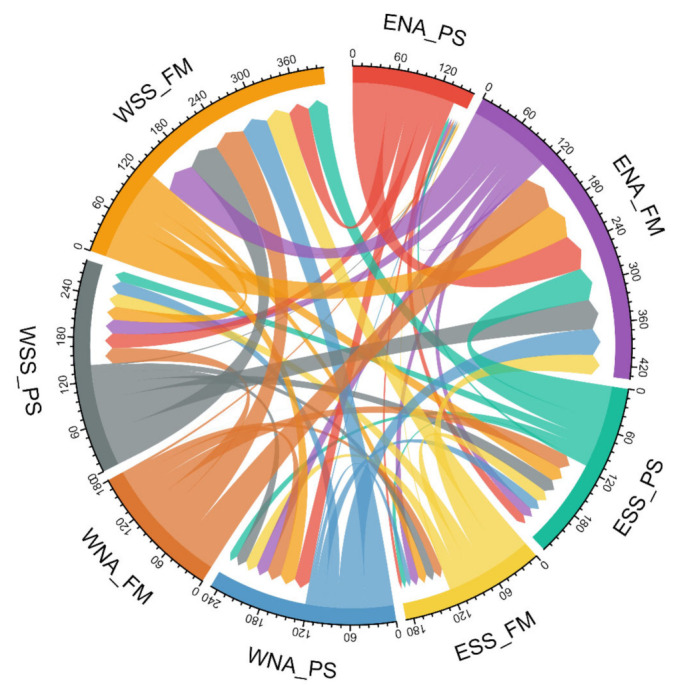
Circular plot of migration/gene flows resulting from the Bayesian coalescent approach in Migrate-n. Numbers around the perimeter illustrate values of 2 Nm = Ɵ × M (the number of immigrants). The arrow signs and their width represent the direction and extent of gene flows between specified groups (ENA_PS: Eastern North African pastoralists; ENA_FM: Eastern North African farmers; WNA_PS: Western North African pastoralists; WNA_FM: Western North African farmers; ESS_PS: Eastern Sahel/Savannah pastoralists; ESS_FM: Eastern Sahel/Savannah farmers; WSS_PS: Western Sahel/Savannah pastoralists; WSS_FM: Western Sahel/Savannah farmers).

When evaluating signals of gene flow obtained from the Bayesian coalescent approach in Migrate-n software (see Supplementary Tables S7 and S8 and Figure 5), we detected the highest population size parameters in farmer populations from eastern North Africa and western Sahel/Savannah belt, which implies larger N_e_ in these groups. Moreover, we calculated the immigration rates and the numbers of immigrants into specific groups. Surprising results are seen in North African populations (in particular eastern pastoralists and western farmers), who seem to have received the least number of immigrants from other regions. On the other hand, we ought to consider the differences in sample sizes of these groups: for eastern North African pastoralists, we had just 195 samples, while for western North African farmers we had 2343. The results also suggest a higher gene flow into farmer populations in general, with the exception of the eastern Sahel/Savannah belt where it was even lower than in other pastoralist groups. Figure 5 represents the summary of population contacts (gene flow) in a circular plot. In general, this specific analysis showed relatively high migration activities not only along the east–west but also the north–south axis.

**Figure 6:**
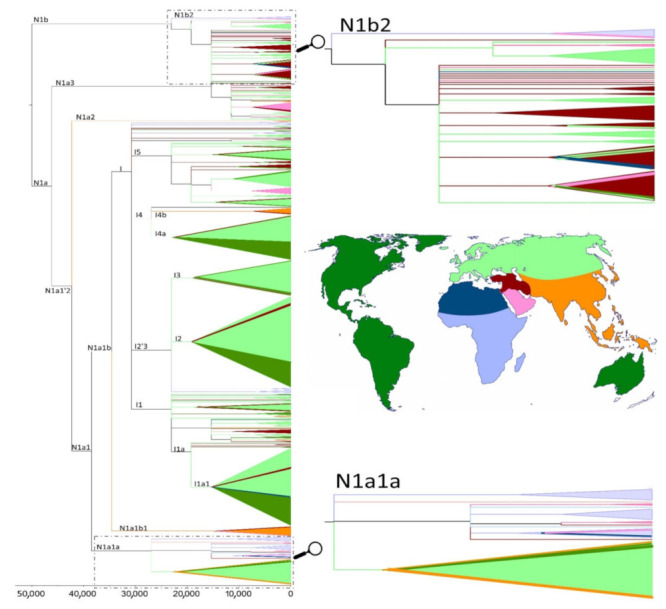
Phylogenetic reconstruction of mtDNA haplogroup N1, with time scale for the most recent common ancestors of the branches. Colors used in the tree correspond to the geographical regions illustrated in the map. Zooms of the N1a1a and N1b2 parts of the tree allow us to observe a closer relationship between the Sahel/Savannah belt and Arabian sequences.

The phylogeny of haplogroup N1, together with its derived clade I, is presented in Figure 6 and Supplementary Figure S1. These figures show that a large proportion of N1 lineages from the Sahel/Savannah belt and from eastern Africa (Somalia, Sudan, Ethiopia, Kenya, and Tanzania), which are affiliated in N1a1a and N1b2 sub-branches, have the closest relatives in the Arabian Peninsula and are the oldest lineages in those sub-branches, while most I-Sahel/Savanah belt lineages are basal in the branches together with other European and Near Eastern sequences. When we look at the age of the N1a1a and N1b2 clusters (Table 2 and Supplementary Table S9), we can see age estimates at most around 20 ka, whereby these should be interpreted as the upper limit age of introgression of these lineages into the Sahel.

## 4. Discussion

An mtDNA dataset containing 7213 mtDNA sequences in 134 African populations—which is much more than used in a previous studies [9,56]—and covering the entire circum-Saharan space had significantly contributed to our understanding of African population history north of the equator. First of all, we were able to show that North African populations have lower values of nucleotide diversity, especially in the western part of the region. This can be attributed to their lower effective population sizes, as attested also by the Bayesian coalescent approach employed in this study. When contrasting pastoralists with farmers, we found similar distributions of diversity values, which supports our previous finding of no significant structure associated with the subsistence strategy in the Sahel/Savannah belt [10].

The lower level of differentiation among the populations of North Africa than among the populations of the Sahel/Sudan belt supports the idea of higher migration activity homogenizing the gene pool and eroding the population structure in the southern Mediterranean space. It is well possible that there was a long-range influx of population(s) from the Near East to the Maghreb already in preagricultural times [50]. In fact, this finds support in recent aDNA analyses of Iberomaurusian skeletons [57]. Interestingly, both Natufian and Iberomaurusian specimens show a high level of Basal Eurasian ancestry, which was a population isolated > 50 ka in a Late Pleistocene refugium of the Arabo-Persian Gulf without contacts with the Neanderthals [58]. Further immigration to North Africa took place in the Neolithic and in later times both from the Near East [59,60,61] and from Europe via the Strait of Gibraltar [62,63,64,65]. According to our results, it seems that this expansion through populations of farmers reached all the way to the western Sahel/Savannah belt. It should also be noted that the general genetic homogeneity of North African populations is reflected in the linguistic homogeneity of Afro-Asian languages.

Our results which suggest that the eastern part of North African populations received immigrants from the Sahel/Savannah belt do not correspond with research on autosomal SNP variants, which had shown that the populations of Egypt and Libya are composed predominantly of a Near Eastern genetic component with very low input from sub-Saharan Africa [50]. However, that may be due to the fact that the last-mentioned study worked with limited sub-Saharan (and not really Sahelian) samples as the putative sources of migration to their North African datasets. In fact, a subsequent study revealed in some North African populations (e.g., in Algeria) a higher gene flow from the sub-Saharan space, especially in maternal lineages [66]. In Egypt, the importance of a migration corridor via the Nile Valley was described a number of times both in archaeology [67] and in genetics [68,69]. Moreover, many eastern Sahel/Savannah populations also have an admixture of sub-Saharan and Eurasian ancestries; especially the Arab groups have an important Eurasian component. This is thus why the sub-Saharan input in North Africa is larger in the west than in the east. Another point is that the sub-Saharan influence in North Africa was mainly via maternal lines: higher in mtDNA, almost nonexistent in the Y-chromosome, and intermediate in the autosomal DNA [70].

Migrations in the Sahel/Savannah belt were probably less important than in North Africa. When we look at the continent-wide African mtDNA diversity, the Sahel/Savannah belt can be viewed as a corridor between the Sahara and tropical rainforests which connects eastern and western Africa but also has—especially in the Lake Chad Basin—some distinctive genetic features [11]. Food-producing strategies came to play an important role in demographic expansions in this region later than in North Africa, especially in the Holocene. The first expansion may have been related to pastoralism, which is a strategy perfectly well-adapted to Sahelian dryland ecosystems [71]. It spread through the Sahel/Savannah belt from northeastern Africa during the Holocene [72,73,74]. An expansion of herders started ~8 ka in northeastern Africa but was relatively slow because it reached the western Sahel/Savannah belt much later, at about 3 ka [75].

The cultivation of cereals and tubers, which is autochthonous in the Sahel/Savannah belt (especially in and around the Middle Niger Delta [76,77,78,79] and in the Middle Nile Valley [80]) was somewhat delayed because the first fully domesticated plants were consumed in Africa only about 4.5 ka. At present, the majority of Sahelian economics is based on mixed agro-pastoralism [81] but in many Sahelian countries, we still find purely nomadic pastoralists. We can thus see that a somewhat delayed spread of a particular food-production subsistence strategy may result in a lower migration activity and higher population differentiation. Despite some morphological differences detected between present-day full-time nomadic pastoralists and sedentary farmers [82], our genetic analyses show that lifestyle cannot be considered a determinative parameter of population sub-structuring in Africa, at least when considering the entire circum-Saharan region and not just the Sahel/Savannah belt where the biological separation of lifestyles may have played a more important role.

It seems that periodically, both in a long-term and short-term view, an increase in the size of the shallow Lake Chad in the middle of the east-western Sahelian corridor presented an obstacle to gene flow, forming a cul-de-sac. This can be documented by the spread of two different populations of nomadic pastoralists: in the west the Fulani, such as Woɗaaɓe, and in the east the Arabs, such as Baggara or Shuwa. While the Arabs are of Eurasian (Arabian) ancestry and received gene flow from sub-Saharan Africans [28], the Fulani are of western African ancestry and their ancestors acquired some Eurasian ancestry by admixture with a northern African population possibly related to the Berbers [24,27]. Since the level of this admixture is relatively high (analyses show around 20% of a Eurasian component), it might be responsible for the noticeable differences between local Fulani populations and the surrounding relatively homogeneous Sahel/Savannah gene pool. On the other hand, the genetic diversity of sedentary farmers suggests that they lived in reproductively more isolated groups, which led to genetic drift and isolation by distance [10]. Interestingly, this can be associated also with the genetic diversity of their pearl millet landraces grown by different ethnolinguistic groups, especially in the western part of the Lake Chad Basin [83].

Finally, we found that the N1 haplogroup, which diversified in southwestern Asia some 55 ka [4] and its younger lineages expanded across all Eurasia, is present also in eastern Africa and the Sahel/Savannah belt. On the other hand, the age estimates of N1 mitogenomes we detected in our Sahelian populations are much younger and thus congruent with population contacts via Ba el-Mandeb or the Red Sea [84], not via the southern Mediterranean space. Moreover, two ancient samples from Takarkori rock shelter in Libya dated to ~7 ka [33] are highly distinct from our N1 Sahelian and eastern African samples because they branched off before all the current N lineages (far away from N1). We did not find any traces of local expansion of mtDNA N1a lineages into the Sahel/Savannah belt, as described for example for the Y chromosome R1b-V88 haplogroup [85,86] or for L3f3 mtDNA haplogroup [15]. This kind of (maternal) Eurasian N1 impact is visible mainly in eastern Africa, especially in Sudan, Somalia, Ethiopia, Kenya, and Tanzania, and not further in the central or even western part of the Sahel/Savannah belt close to the Lake Chad Basin. In fact, migration associated with this N1 African diversity might be associated with the Late Pleistocene/Holocene expansions in Arabia and the neighboring region and more recently also with the spread of Ethiosemitic languages into Ethiopia at ~3 ka (for example N1a3a+195C!), which continued further to South Africa [87] and not to the west.

## Figures and Tables

**Table 1:** Kruskal–Wallis one-way test (*p*-values) of differences in average diversity indices and demographic parameters among specified groups of populations.

	Kruskal–Wallis Test (*p*-Values)					
	Haplotype Diversity	Nucleotide Diversity	Mean Number of Pairwise Differences	Fu’s Fs (*p*)	Hri (*p*)	SSD (*p*)
NA vs. SS	0.380	**0.000**	**0.000**	0.563	0.068	0.208
FM vs. PS	**0.004**	0.856	0.630	0.133	0.271	0.249
AA vs. NC vs. NS	0.293	**0.011**	**0.001**	0.682	**0.033**	0.147
eNA vs. eSS vs. wNA vs. wSS	0.453	**0.000**	**0.000**	0.940	0.266	0.231
NAFM vs. NAPS vs. SSFM vs. SSPS	**0.000**	**0.000**	**0.000**	0.507	0.482	0.874

NA: North Africa; SS: Sahel/Savannah belt; FM: farmers, PS: pastoralists; AA: Afro-Asiatic; NC: Niger-Congo; NS: Nilo-Saharan; eNA: eastern North Africa; eSS: eastern Sahel/Savannah; wNA: western North Africa; wSS: western Sahel/Savannah; NAFM: farmers North Africa; NAPS: pastoralists North Africa; SSFM: farmers Sahel/Savannah; SSPS: pastoralists Sahel/Savannah; *p*-values marked with bold indicate statistically significant differences between the groups.

**Table 2:** Age estimates and standard deviations (in years) for N1a haplogroups in Africa.

	Maximum Parsimony				Maximum Likelihood	
	Total Clock		Synonymous Clock		All SNPs	
Haplogroup	Age	95% CI	Age	95% CI	Age	95% CI
N1a1a	24,005	16,482–31,778	20,727	10,873–30,580	21,754	21,304–22,182
N1a1a3	13,900	10,071–17,808	14,266	7442–21,090	17,948	17,676–18,211
N1a3a+195C!	3188	1266–5135	1819	−838–4477	3610	3577–3643
N1b2+9325C	6816	553–13,327	14,191	−3 562–31,944	6908	6762–7048

## Data Availability

The sequences were submitted to GenBank and are available under accession numbers OM952925-OM953385 for HVS-1 sequences and OM990709-OM990737 for mitogenomes.

## Supplementary Materials

The following supporting information can be downloaded at: https://www.mdpi.com/article/10.3390/genes13030533/s1, Supplementary Table S1: General information on studied population samples. Supplementary Table S2: Samples used for mitogenome sequencing. Supplementary Table S3: Samples used for phylogenetic analyses of the N1 haplogroup. Supplementary Table S4: The results of intrapopulation analyzes. Supplementary Table S5: Analyses of molecular variance (AMOVA). Supplementary Table S6: Matrix of coancestry coefficients colored according to genetic distance between populations in green < 0.09; yellow < 0.19; red > 0.2. Supplementary Table S7: Estimation of population size parameters scaled to mutation rate based on coalescent Bayesian approach Migrate-n. Supplementary Table S8: Estimation of immigration rate (A) and calculation of the relative number of immigrants (B) based on the results of coalescent Bayesian approach Migrate-n. Supplementary Table S9: Age estimates and standard deviations (in years) for the TMRCA of all N1a haplogroups analyzed. Supplementary Figure S1: The derived clade I of mtDNA haplogroup N1.
